# “It’s a horrible assignment”: A qualitative study of labor and delivery nurses’ experience caring for patients undergoing labor induction for fetal anomalies or fetal demise

**DOI:** 10.1016/j.contraception.2021.04.014

**Published:** 2021-04-22

**Authors:** Blake Zwerling, Julie Rousseau, Kelly Marie Ward, Ellen Olshansky, Alyssa Lo, Heike Thiel de Bocanegra, Tabetha Harken

**Affiliations:** a University of California, Irvine Department of Obstetrics and Gynecology, Orange, CA, United States; b University of California, Irvine Sue & Bill Gross School of Nursing, Irvine, CA, United States; c University of Wisconsin – Madison, Departments of Gender & Women’s Studies and Sociology, Madison, WI, United States

**Keywords:** Abortion, Induction termination, IUFD, Nurses, Qualitative research, Thematic analysis

## Abstract

**Objectives:**

This study sought to explore labor and delivery (L&D) nurses’ experiences caring for women undergoing induction for intrauterine fetal demise (IUFD) or termination for fetal anomalies, and to characterize reluctance towards participation in abortion care or – conversely – the commitment to provide services.

**Study Design:**

Researchers conducted a qualitative study that consisted of open-ended, semistructured interviews with 15 registered nurses who care for women on L&D at a large metropolitan hospital. We analyzed these data for content and themes.

**Results:**

Labor and delivery nurses struggle emotionally, logistically, and morally with bereavement care, whether their patients are experiencing an IUFD or termination for fetal anomalies. The analysis generated the following themes: the emotionally intense work of perinatal loss, feelings of incompetence in bereavement care, ethical conflicts, and judgment of both termination and IUFD patients. In addition, nurses who chose to provide care for patients undergoing induction termination for fetal anomalies described a duty to care for all patients despite the increased logistic and emotional burden.

**Conclusions:**

Much of the discomfort L&D nurses reported caring for patients undergoing induction termination stems from the emotional toll, lack of skills, and bureaucratic burden of bereavement care rather than a moral objection to abortion. Instituting interventions to improve staffing, simplify paperwork, augment bereavement training, and improve support for the emotional burden of caring for these patients may therefore increase access to competent and compassionate abortion care.

**Implications:**

Labor and delivery nurses struggle with bereavement care whether their patients are experiencing an IUFD or termination for fetal anomalies. Instituting interventions – like interdisciplinary simulation – to support nurses in bereavement care may increase the number willing to participate in abortion care, thereby improving patient access.

## 1. Introduction

Popular portrayals of Labor and Delivery (L&D) units focus on happy outcomes, but this ignores the reality that some patients experience perinatal fetal loss through intrauterine fetal demise (IUFD, defined as fetal loss at or after 20 weeks gestational age) or induction termination for fetal anomalies or other obstetric complications. In the United States (US), providers generally provide labor inductions for IUFDs or second trimester abortion in the inpatient setting (typically on an L&D unit) employing prostaglandin analogues to induce uterine contractions and the expulsion of all products of conception. These procedures are relatively common: in the US, 5.96 IUFDs occurred for every 1000 live births in 2013 [1]. Additionally, though precise US rates are not available, Kerns et al. [2] estimated that 47% to 90% of all anomalous pregnancies end in termination [2].

Data on the attitudes and experiences of nurses – specifically L&D nurses – caring for patients undergoing second trimester induction termination for fetal anomalies are scant. Overall, nurses’ attitudes towards abortion have not been sufficiently explored [3,4]. Views of abortion care vary by nationality, professional title, experience in abortion care, personal obstetric history, religious beliefs, reason for termination, and gestational age of the fetus (though none of these categories confer uniform opinions) [5]. McLemore, et al. [6] qualitatively described and analyzed the cognitive, emotional, and behavioral processes nurses use to decide whether to participate in abortion care. However, few other studies have explored the basis of these views or whether the hesitancy to participate in care stems from opposition to abortion versus a discomfort with perinatal loss generally. Specific factors such as personal experience and commitment to patient autonomy that drive nurses to accept these assignments are incompletely understood.

In the US, abortion care is voluntary for all healthcare professionals with legal protections for conscientious objectors [7]. The ability to opt-out decreases the number of available nurses and increases the burden for those who participate.

In non-US practice environments such as Australia, nurses participating in abortion care report high rates of secondary trauma leading to emotional and physical exhaustion that may further deplete the workforce [8]. The source of nurses’ stress and discomfort in caring for patients choosing induction terminations is unclear. We must learn more about the experiences of these nurses in order to assure sustainable provision of competent and compassionate nursing care for women experiencing IUFDs and induction terminations. The aims of this study were to qualitatively assess the attitudes of L&D nurses caring for women undergoing labor induction for IUFD or fetal anomalies. Specifically, the study sought to parse moral objection to abortion care versus general unease with perinatal loss.

## 2. Materials and methods

### 2.1. Study design, sampling, and recruitment

We conducted a qualitative description of interviews of 15 nurses from an L&D unit at an academic tertiary care research hospital with a level three neonatal intensive care unit serving a region of approximately three million people in Southern California [9]. The hospital cares for a disproportionate number of complicated cases compared to other regional hospitals. We used purposive sampling to identify potential participants. Author JR led recruitment by sending emails to all eligible nurses and placing recruitment flyers in breakrooms. Interested nurses then contacted KW, the member of the research team who conducted the interviews. Recruitment began after obtaining approval under the University of California, Irvine IRB.

### 2.2. Interview guide

Prior to recruitment, the research team developed a list of broad questions and possible follow-up questions for a semistructured interview guide. The interviewer followed the guide for details and clarification as needed. In general, the interview covered the following topics: respondents’ professional trajectories; education and training regarding perinatal loss; caring for patients experiencing perinatal loss; personal beliefs regarding perinatal loss and pregnancy termination; and organizational characteristics of their unit. One interviewer – KW, a sociologist by training – conducted all interviews, providing consistency in data collection. Interviews lasted 45 to 120 minutes and occurred outside of work at a date, time, and location chosen by the respondent. After audio recording the interviews, the authors then transcribed, and uploaded them to ATLAS.ti. The authors provided each participant with a gift card for $30 after the interview.

### 2.3. Data analysis

Authors BZ, AL, and TH conducted thematic analysis of the data [10]. The coding team based initial coding on the broad themes from the guide while also recording emergent themes. The coding team met to discuss their initial findings, received feedback from the research team, developed a more comprehensive coding rubric, and again applied the revised rubric to the data. In presentation of the data, nurses are identified by pseudonym followed by years in practice e.g. (Sophie, 8).

## 3. Results

KW conducted interviews with 15 L&D nurses. Nurses in the sample were all women and ranged from having one to 38 years of experience as a Registered Nurse, with a median of ten years. They reported having worked between one and 14 years at the field site with a median of 5.5 years. Accordingly, participants reflect a wide range of time as nurses and at the hospital under study.

The 15 L&D nurses reported similar challenges caring for women experiencing IUFDs and induction termination. The following salient themes emerged: (1) the emotionally intense work of perinatal loss; (2) feelings of incompetence in bereavement care; (3) the burden of the willing; (4) ethical conflicts and judgment. Specific to induction termination care, many spoke of their (5) religiosity both as a reason to recuse themselves and as a driver to participate in the work.

### 3.1. Emotionally intense work of perinatal loss

When working with both IUFD and induction termination patients, most reported a high-level of emotional distress compounded by the fact that they had selected L&D work specifically anticipating positive birthing experiences: “I probably had no idea in the beginning before choosing [L&D]” (Maria, 14). In particular, handling fetuses seemed to drive home reality: “We’re hands on with them…it’s graphic. It’s really graphic…And I think that’s the hardest part” (Leticia, 2.5).

Nurses noted that both patient populations required intense emotional support: “It almost feels like you have to wear another hat of therapist too…I need to be present with this patient and I need to kind of support them emotionally too and not just try and do all my tasks that a normal nurse would do” (Leticia, 2.5). Nurses often felt responsible for tempering trauma perpetuated on the patients by other care providers: “The physician just walks in…and they drop the bomb and walk out…So the nurse is left picking up all of the pieces and trying to comfort the patient. And it takes a toll on you too. You’re the one sitting there watching…Over and over and over” (Roberta, 25).

Many nurses described the personal emotional burden of the care. One reported: “I need to talk about this because clearly it’s eating away at me and having an effect on me and I’m not handling it” (Lana, 5). Many internalized their patients’ suffering to some degree: “Let’s say you and me are work buddies…I would be like…you’ll never guess what happened to me today. It’s not what happened to the patient. It’s what happened to me” (Linda, 5).

Nurses employed different strategies to maintain boundaries and to manage their own conflicting, complicated emotions. “We have to emotionally remove ourselves from the situation. Which sounds so terrible… you have to make sure that the mom is still stable…Or making sure that everything is okay that you just forget to be present emotionally” (Leticia, 2.5) Some interpreted the perimortem tasks as acts of service. One described: “We take photos and footprints…And some of the nurses really love it…it brings them, not joy, but it makes them feel good to be able to provide that service to others” (Maria, 14). In this way, emotional distancing and focusing on the positive impact of their service helped some nurses balance the emotionally difficult work with clinical care duties.

### 3.2. Feelings of inadequacy in bereavement care

Nurses acknowledge the unique set of skills necessary to support patients through bereavement care: “I don’t think everyone can do it, definitely. Some people are better at it than others” (Laura, 8). L&D nurses reported receiving inadequate preparation during formal training: “We touch on it in nursing school but not a lot and not really specific toward the family, losses of a baby…” (Amy, 10). In contrast to many areas of nursing, the L&D nurses additionally reported limited daily exposure to death and dying, which compounded their unease. “We’re uncomfortable and we don’t know how to deal with this patient’s grief and so we don’t go in the room as often as we should or we’re not really present for the patient as much as we would want to be” (Amy, 10).

Without sufficient education, nurses worried they may inadvertently do harm to patients already experiencing intense suffering: “You say the wrong thing or do the wrong thing…It’s like I’m failing…I want that person to feel like I was there for them and I did a good job and they felt loved or cared for in a proper way” (Carly, 5). Nurses want to do right by their patients and to feel confident and competent in their work, which make feelings of inadequacy around perinatal loss all the more uncomfortable.

Compounding the increased emotional work of bereavement care, unfamiliar and complicated paperwork also increased. “If the baby is born alive, you use the red packet. If the baby is born dead, you use the yellow packet. If the baby is born before 20 weeks, it’s this paperwork. If it’s after 20 weeks, it’s this paperwork” (Lana, 5). Nurses reported multiplying tasks, including creation of memory boxes, taking photographs, and handling of the dead fetus. “Dealing with terminations is very unpleasant. The paperwork you have to do, dealing with the family you have to do. The aftercare you have to do is very unpleasant” (Susan, 12). This increased workload left nurses feeling overly burdened emotionally and logistically.

### 3.3. The burden of the willing

Nurses at the institution are not permitted to opt-out of care for patients experiencing IUFDs, but are – under federal law – legally permitted to conscientiously object to caring for those undergoing elective induction terminations for fetal anomalies [7]. Nurses willing to care for patients choosing induction termination were in the minority. “The one hard thing is there’s very few…there’s like 40 of us day shift nurses and there’s probably eight to ten who will do it. So that’s not a lot if you think about on a day to day basis” (Amy, 10). The opt-out system for induction termination care at the institution unexpectedly at times funneled IUFD cases to the same minority of nurses who participated in abortion care given their increased experience with perinatal loss: “If it’s an IUFD where the baby is demised, there is still like a certain bunch of us that takes care of those patients…It all gets lumped together no matter what…I think technically no…you cannot opt-out of taking care of a demise, but I still feel like it falls on a certain few of us” (Bianca, 2.5).

### 3.4. Ethical conflicts and judgment surrounding bereavement care

The theme of judgment arose throughout the nurses’ interviews and extended to patients and their families, as well as fellow nurses and other care providers. The nurses identified patients’ intense guilt and shame, which was at times reinforced by their families: “She felt so bad…And she still wanted to do it and her sister flew in from a different state just to be with her. Because her husband wouldn’t be with her” (Laura, 8). Nurses themselves judged patients for choosing induction termination at times: “I think we all know people with Downs who are high functioning, lovable, amazing people. The fact that you’re still allowed to terminate for that, I think it would have been really hard and heartbreaking for any nurse having to care for a family that chose [that]” (Lana, 5).

While judgment often centered on patients undergoing induction termination, it was a pervasive theme surrounding patients experiencing IUFDs as well. Nurses expressed frustration at maternal behaviors such as substance abuse, lack of prenatal care, or inadequate management of chronic medical conditions that may have contributed to fetal loss: “We’ve had patients not come in for their appointments, not show up for their scheduled C-sections, and they come in and their baby is dead. And you’re like, we could have prevented this” (Doris, 38).

For those choosing to participate in induction termination care, some referenced a professional duty to care for all patients regardless of circumstances: “Whatever the patient chose, my religious beliefs or how I chose to think of it wasn’t my choice. I’m going to care for my patient and [give] her the best experience because everybody as a patient needs to be taken care of” (Wendy, 10). Others intellectually removed themselves to remain nonjudgmental: “At the end of the day, it’s [the patient] that will have those feelings later on…And not me. So I’m there to take care of you and whatever decision that you are making” (Vanessa, 4.5).

### 3.5. Religious views and abortion care provision

Some nurses cited religious reasons for refusing to participate, but others provided faith-based counter examples that drove them to care for these patients: “I’m going to care for my patients no matter what their choices are…my religious preference…it’s not my choice…So of course I’m not going to get penalized by my religion because I’m caring for the person that’s choosing this” (Wendy, 10). Another nurse added: “I kind of just pray about it and I ask God for forgiveness that I’m part of something that I don’t think is in His will…I just am thankful that Jesus forgave us for whatever we do…I feel like even if I were to just try to protect this patient from maybe what’s going to be something she regrets, I don’t think I could do it in a way where I’m not going to end up making her feel like I’m judging her. So I have to just accept the choice that she made” (Susan, 12). Though religion is often cited as a reason not to participate in abortion care, these examples demonstrate that faith-based values of duty to care and nonjudgement drive others to the work.

Throughout, nurses recognized the difficult balance of personal and professional ethics and responsibilities when deciding whether to participate in the care of patients undergoing induction terminations: “As a nurse, it’s kind of your job to care for whoever comes in the door, but, honestly…if somebody is like very religious, like it’s not fair to ask them to take care of somebody who is doing an induction termination” (Lana, 5). However, at times this engendered resentment: “You might get more than somebody else because they…don’t want to participate in something like that…That I think is not the most fair. Because why do I have to have all of these assignments that are more emotionally draining because I’m open-minded and not judgmental and not religious” (Roberta, 25). Some questioned the motives for refusing patients undergoing induction terminations: “Some people, I’m not even sure if they opt-out because they really religiously feel that way or simply because you can opt-out, because it’s a horrible assignment” (Susan, 12). These quotes illustrated the myriad of personal, professional, and religious considerations nurses weighed in deciding whether to care for patients having an induction termination.

## 4. Discussion

Previous studies have shown that nurses have a less favorable attitude toward abortion than other healthcare professionals [5]. Nurse ambivalence to participate in abortion care has been shown to negatively affect patients and to block access to care [11]. Our data show that nurses struggle emotionally, functionally, logistically, and morally caring for women undergoing induction of labor for fetal demise as well as fetal anomalies. Creating effective interventions and structural changes depends on better understanding the drivers of L&D nurse discomfort.

Previous studies have shown that nurses had feelings of personal failure and helplessness with grieving patients and many desired skill-building through continue education and workshops [12–15]. These data similarly demonstrate that L&D nurses report lack of confidence in their skills, leading to avoidance of perinatal loss patients. McLemore, Levi, and James [16] found that improving training and support of nurses working in abortion can increase nurses’ support and improve retention. Hewitt and Cappiello [17] also described essential competencies in nursing education for care of unintended pregnancy. Interventions have been described in areas of nursing (e.g., intensive care units) with more common bereavement components that explore mechanisms for limiting and treating burnout from these emotionally challenging scenarios. In abortion care, the provider share workshops described by Harris et al. [18,19] have shown the benefits of narratives, art, and facilitated sharing sessions for the emotional benefit of abortion providers as well.

Strengths of this study included the in-depth interview format, which provides a window into the real lived experiences of individuals. Given the qualitative format, the study could explore the ‘why’ behind personal decisions to participate in induction termination care and sought to further explore what emanated from personal discomfort due to moral and/or religious objection to abortion versus unease with perinatal loss generally.

As an interdisciplinary group, the research team reflects a broad range of academic and clinical backgrounds. While overall this is a strength in scholarship, we recognize potential limitations. Having an outsider (a sociologist and nonclinician) conduct interviews was helpful so that respondents could speak freely about their experiences. On the other hand, it is possible the interviewer’s lack of clinical expertise resulted in missed opportunities to probe for certain kinds of information during interviews.

In addition, it is possible the team prematurely terminated the enrollment of participants, with respondents not accurately reflecting the extent of variation on the unit, leading to missed additional/new information. Our recruitment strategy relied on people contacting the interviewer, thus making it likely that only people who were comfortable talking about perinatal loss volunteered to participate. These data may not reflect those nurses who were ambivalent or uncomfortable talking about the topic. Thus, our findings cannot extend to other inpatient L&D units. However, given how cases of perinatal loss and induction termination are allocated in the unit, it is likely that those who are comfortable with talking about the topic are also those who are disproportionately assigned those cases. As we were interested in the experiences of nurses who provide this type of care, it follows that we would seek out those who have more experience.

These data illuminate unique challenges facing L&D nurses who care for these patients. We propose that elucidating their experiences may aid in providing targeted trainings that could alleviate much of the burden of this care in the hopes of increasing retention and improving quality of care. Though some nurses seemed to have chosen the field, in part, for the happy moments of live births with healthy moms and infants, many recognized the enhanced potential to ease suffering in patients experiencing perinatal loss that went above and beyond their typical assignments. However, providing this care comes at a cost. The time has come to recognize the expertise required to excel in caring for patients undergoing labor induction for IUFD or fetal anomalies and to commit to ongoing education, training, and support.

## References

[1] MacDorman MF, Gregory ECW. Fetal and perinatal mortality: United States, 2013. Natl Vital Stat Rep 2015;64:1–24. 26222771

[2] Kerns JL, Swanson M, Pena S, Wu D, Shaffer BL, Tran SH, Characteristics of women who undergo second-trimester abortion in the setting of a fetal anomaly. Contraception 2012;85:63–8. 22067803 10.1016/j.contraception.2011.04.012

[3] Marshall SL, Gould D, Roberts J. Nurses’attitudes towards termination of pregnancy. J Adv Nurs 1994;20:567–76. 7963066 10.1111/j.1365-2648.1994.tb02397.x

[4] McLemore M, Levi A. Nurses and care of women seeking abortions, 1971 to 2011. J Obstet Gynecol Neonatal Nurs 2011;40:672–7. 10.1111/j.1552-6909.2011.01302.x 22273447

[5] Lipp A A review of termination of pregnancy: prevalent health care professional attitudes and ways of influencing them. J Clin Nurs 2008;17:1683–8. 18592622 10.1111/j.1365-2702.2007.02205.x

[6] McLemore MR, Kools S, Levi AJ. Calculus formation: nurses’ decision-making in abortion-related care. Res Nurs Heal 2015;38:222–31. 10.1002/nur.21655 25820100

[7] Conscience protections for health care providers | HHS.gov n.d. https://www.hhs.gov/conscience/conscience-protections/index.html (accessed August 6, 2020).

[8] Lipp AJ, Fothergill A. Nurses in abortion care: identifying and managing stress. Contemp Nurse 2009;31:108–20. 19379113 10.5172/conu.673.31.2.108

[9] Sandelowski M Focus on research methods: whatever happened to qualitative description? Res Nurs Heal 2000;23:334–40. 10.1002/1098-240x(200008)23:4<334::aid-nur9>3.0.co;2-g 10940958

[10] Braun V, Clarke V. Using thematic analysis in psychology. Qual Res Psychol 2006;3:77–101.

[11] Henshaw S Factors hindering access to abortion services. Fam Plann Perspect 1995;27:54–9 87. 7796896

[12] Chan MF, Wu LH, Day MC, Chan SH. Attitudes of nurses toward perinatal bereavement: findings from a study in Hong Kong. J Perinat Neonatal Nurs n.d;19:240–52. 10.1097/00005237-200507000-00010 16106232

[13] Hutti MH, Polivka B, White S, Hill J, Clark P, Cooke C, Experiences of nurses who care for women after fetal loss. J Obstet Gynecol Neonatal Nurs 2016;45:17–27. 10.1016/j.jogn.2015.10.010 26815795

[14] Puia DM, Lewis L, Beck CT. Experiences of obstetric nurses who are present for a perinatal loss. J Obstet Gynecol Neonatal Nurs 2013;42:321–31. 10.1111/1552-6909.12040 23682697

[15] McCreight BS. Perinatal grief and emotional labour: a study of nurses’ experiences in gynae wards. Int J Nurs Stud 2005;42:439–48. 15847906 10.1016/j.ijnurstu.2004.07.004

[16] McLemore MR, Levi A, James EA. Recruitment and retention strategies for expert nurses in abortion care provision. Contraception 2015;91:474–9. 25708505 10.1016/j.contraception.2015.02.007 PMC4442037

[17] Hewitt C, Cappiello J. Essential competencies in nursing education for prevention and care related to unintended pregnancy. JOGNN - J Obstet Gynecol Neonatal Nurs 2015;44:69–76. 25580525 10.1111/1552-6909.12525

[18] Debbink MLP, Hassinger JA, Martin LA, Maniere E, Youatt E, Harris LH. Experiences with the Providers Share Workshop Method: abortion worker support and research in tandem. Qual Health Res 2016;26:1823–37. 27496534 10.1177/1049732316661166

[19] Mosley EA, Martin L, Seewald M, Hassinger J, Blanchard K, Baum SE, Addressing abortion provider stigma: a pilot implementation of the providers share workshop in sub-saharan Africa and latin America. Int Perspect Sex Reprod Health 2020;46:35–50. 10.1363/46e8720 32375117

